# Effects of high-intensity interval training, moderate-intensity continuous training, and guideline-based physical activity on cardiovascular metabolic markers, cognitive and motor function in elderly sedentary patients with type 2 diabetes (HIIT-DM): a protocol for a randomized controlled trial

**DOI:** 10.3389/fnagi.2023.1211990

**Published:** 2023-08-15

**Authors:** Peng Yu, Zicheng Zhu, Jiahui He, Bohua Gao, Qi Chen, Yifan Wu, Jing Zhou, Yixuan Cheng, Jitao Ling, Jing Zhang, Ao Shi, Huijing Huang, Runlu Sun, Yan Gao, Weiguang Li, Xiao Liu, Zhiwei Yan

**Affiliations:** ^1^Department of Endocrinology and Metabolism, The Second Affiliated Hospital of Nanchang University, Nanchang, Jiangxi, China; ^2^Institute for the Study of Endocrinology and Metabolism in Jiangxi Province, Nanchang, Jiangxi, China; ^3^The Second Clinical Medical College of Nanchang University, Nanchang, Jiangxi, China; ^4^School of Basic Medicine, Fujian Medical University, Fuzhou, China; ^5^Laboratory of Exercise Physiology, Liaoning Province Sports Development Center, Shenyang, Liaoning, China; ^6^Department of Anesthesiology, The Second Affiliated Hospital of Nanchang University, Nanchang, Jiangxi, China; ^7^St. George’s University of London, London, United Kingdom; ^8^School of Medicine, University of Nicosia, Nicosia, Cyprus; ^9^Department of Cardiology, Sun Yat-sen Memorial Hospital of Sun Yat-sen University, Guangzhou, Guangdong, China; ^10^Guangdong Province Key Laboratory of Arrhythmia and Electrophysiology, Guangzhou, Guangdong, China; ^11^College of Kinesiology, Shenyang Sport University, Shenyang, Liaoning, China; ^12^Cardiology Department, Liaoning Province Jinqiu Hospital, Shenyang, Liaoning, China; ^13^Provincial University Key Laboratory of Sport and Health Science, School of Physical Education and Sport Sciences, Fujian Normal University, Fuzhou, Fujian, China

**Keywords:** aging, sedentary behavior, high-intensity interval training, cardiovascular health, cognitive function, motor function, physical activity

## Abstract

**Background and objective:**

Sedentary behavior is of increasing concern in older patients with type 2 diabetes mellitus (T2DM) due to its potential adverse effects on cardiovascular health, cognitive function, and motor function. While regular exercise has been shown to improve the health of individuals with T2DM, the most effective exercise program for elderly sedentary patients with T2DM remains unclear. Therefore, the objective of this study was to assess the impact of high-intensity interval training (HIIT), moderate-intensity continuous training (MICT), and guideline-based physical activity programs on the cardiovascular health, cognitive function, and motor function of this specific population.

**Methods:**

This study will be a randomized, assessor-blind, three-arm controlled trial. A total of 330 (1:1:1) elderly sedentary patients diagnosed with T2DM will be randomly assigned the HIIT group (10 × 1-min at 85–95% peak HR, intersperse with 1-min active recovery at 60–70% peak HR), MICT (35 min at 65–75% peak HR), and guideline-based group (guideline group) for 12 weeks training. Participants in the guideline group will receive 1-time advice and weekly remote supervision through smartphones. The primary outcomes will be the change in glycosylated hemoglobin (HbA1c) and brain-derived neurotrophic factor (BDNF) after 12-weeks. Secondary outcomes will includes physical activity levels, anthropometric parameters (weight, waist circumference, hip circumference, and body mass index), physical measurements (fat percentage, muscle percentage, and fitness rate), cardiorespiratory fitness indicators (blood pressure, heart rate, vital capacity, and maximum oxygen), biochemical markers (high-density lipoprotein, low-density lipoprotein, triglycerides, total cholesterol, and HbA1c), inflammation level (C-reactive protein), cognitive function (reaction time and dual-task gait test performance), and motor function (static balance, dynamic balance, single-task gait test performance, and grip strength) after 12 weeks.

**Discussion:**

The objective of this study is to evaluate the effect of 12 weeks of HIIT, MICT, and a guideline-based physical activity program on elderly sedentary patients diagnosed with T2DM. Our hypothesis is that both HIIT and MICT will yield improvements in glucose control, cognitive function, cardiopulmonary function, metabolite levels, motor function, and physical fitness compared to the guideline group. Additionally, we anticipate that HIIT will lead to greater benefits in these areas. The findings from this study will provide valuable insights into the selection of appropriate exercise regimens for elderly sedentary individuals with T2DM.

**Ethics and dissemination:**

This study has been approved by the Ethics Review Committee of the Reproductive Hospital Affiliated with China Medical University (approval number: 202203). Informed consent will be obtained from all participants or their guardians. Upon completion, the authors will submit their findings to a peer-reviewed journal or academic conference for publication.

**Clinical trial registration:**

Chinese Clinical Trial Registry, identifier ChiCTR2200061573.

## Introduction

Worldwide, population aging has led to a rise in the number of elderly individuals living with chronic illnesses. Among these illnesses, type 2 diabetes mellitus (T2DM) is a prevalent metabolic condition, with prevalence rates projected to double in the next three decades, posing substantial medical and economic risks (Cho et al., 2018). Furthermore, T2DM often coexists with cardiovascular disease (CVD), with a staggering eight out of ten T2DM patients succumbing to CVD-related complications (Laakso, 2001). In addition to cardiovascular risks, individuals with T2DM may also experience a decline in motor and cognitive function (Chohan et al., 2021; Farup et al., 2021). They exhibit worse performance in terms of standing balance and walking speed, rendering them more susceptible to falls (Morrison et al., 2012; Volpato et al., 2012). Moreover, the behavior of patients could be affected by diseases, and sedentary behavior is becoming increasingly common. Elderly individuals are more likely to exhibit a sedentary lifestyle, which significantly contributes to the rising prevalence of T2DM (Farup et al., 2021). A sedentary lifestyle can result in various adverse effects, such as a decline in cardiopulmonary fitness (Chohan et al., 2021), elevated cardiovascular risk, cognitive decline, and impaired motor function (Morrison et al., 2012). Consequently, the combination of a sedentary lifestyle and advanced age increases the susceptibility of T2DM patients to concurrent cardiovascular impairment and cognitive and motor dysfunction.

Numerous non-pharmacological interventions (diet manipulation, exercise, etc.) have been used for the prevention and management of type 2 diabetes. Regular exercise is widely recognized as an effective strategy for both the prevention and management of T2DM (Volpato et al., 2012). The World Health Organization 2020 guidelines on physical activity and sedentary behavior provide an overview of exercise that is beneficial for elderly individuals and suggest moderate-intensity continuous training (MICT) at least 3 days a week (World Health Organization, 2020). Substantial evidence from clinical and experimental research shows that MICT has a variety of benefits in patients with T2DM, including protection of cardiovascular health, improvement of cognitive function and fall prevention (Morrison et al., 2014; Chiang et al., 2019). Moreover, there is a growing body of evidence suggesting that high-intensity interval training (HIIT) presents a time-efficient alternative to MICT. The interaction between exercise intensity and duration plays a pivotal role in eliciting distinct adaptations, but the optimal model for exercise remains uncertain. Therefore, comparing HIIT and MICT is crucial in the context of T2DM rehabilitation. Research has indicated that HIIT yields comparable, if not superior, cardiovascular benefits for individuals with T2DM. However, importantly, the available evidence regarding its efficacy in elderly sedentary patients with T2DM is still limited (de Mello et al., 2022; Li et al., 2022). Furthermore, there are insufficient data to draw definitive conclusions about the impact of HIIT and MICT on cognitive and motor function in this specific population. While HIIT holds promise as an alternative to MICT, particularly in terms of cardiovascular benefits, its application and suitability for elderly sedentary patients with T2DM require further investigation. Additionally, more research is needed to fully understand the effects of both exercise modalities on cognitive and motor function in this specific population. Such investigations will provide valuable insights into optimizing exercise prescriptions for individuals with T2DM, promoting their overall wellbeing and functional capacity.

Therefore, we designed a single-blind randomized controlled trial to analyze the effects of HIIT, MICT, and guideline-based physical activity on cognitive function, motor function, cardiovascular health, and metabolic markers in sedentary elderly patients with T2DM.

## Materials and methods

### Research design

**FIGURE 1:**
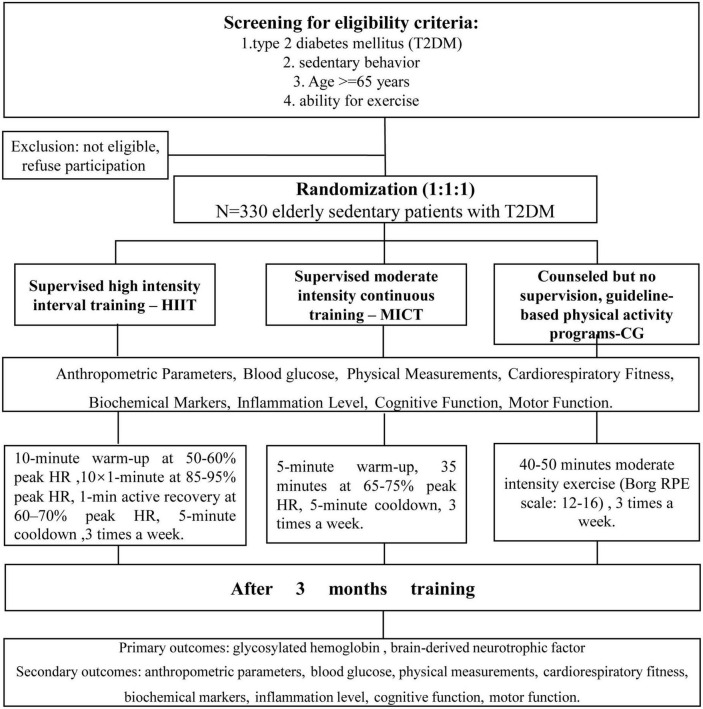
Flowchart of participant recruitment and study procedures. Eligible subjects will be randomly assigned to the HIIT group, the MICT group, or the guideline group and evaluated at two time points. HIIT, high-intensity interval training; MICT, moderate-intensity continuous training.

This study is a randomized, assessor-blind, three-arm, superiority, parallel controlled trial. Participants will be randomly assigned to the HIIT group, MICT group, or guideline group. Testing will be performed at baseline and after the 12-week intervention. The minimum attendance rate will be established in light of the least beneficial dose of exercise. According to previous research (Abreu et al., 2021), participants who complete fewer than 24 training sessions will be considered to drop out of the study. All health and fitness tests will be conducted using the same equipment at the different testing sites. All blood samples will be measured by the same type of facility, and all blood samples will be stored in the Experimental Center of China Medical University for subsequent analysis. An overview of the trial procedures is displayed in Figure 1. This study was approved by the Ethics Review Committee of the Reproductive Hospital Affiliated to China Medical University (approval no. 202203, China clinical trial registration number: ChiCTR2200061573^1^). All patients will provide written informed consent prior to enrollment, and the results of the study will be reported following the Consolidated Standards of Reporting Trials (CONSORT) guidelines.

### Research setting

We will recruit 330 eligible T2DM participants from 3 communities in Shenyang by posters, leaflets and brochures and by conducting health seminars; community physicians will be involved in participant recruitment. All diagnostic participants will be evaluated by clinicians. The exercise interventions and measurements of anthropometric parameters and physiological indicators will be performed at community exercise clinics. The research staff who blinded to group allocation will collect baseline data, supervise the training, and finish outcomes related test in clinical exercise physiology. Participants was initial enrolled in June 2022 and the recruitment is still undergoing.

### Inclusion criteria

Participants will be included for both of the following criteria: (1) self-reported physical activity of < 150 min per week of moderate-intensity for the past 6 months, (2) accelerometer measured energy consumption of ≤ 1.5 metabolic equivalents (METs) according to previous study of Ku et al. (2017). Briefly, participants will wear the accelerometers continuously for 7 days (5 weekdays and 2 weekend days, excepting bedtime). The average energy consumption will be calculated with a requirement of for a minimum of 10 h per day of 7 days. (3) Have T2DM and regularly take oral hypoglycemic drugs, (4) age ≥ 65 years, (5) consciousness and ability to volunteer for exercise intervention, (6) lack a self-reported hypertension diagnosed by physician or hypertension with well-controlled blood pressure (< 140/90 mmHg).

### Exclusion criteria

Patients who meet any of the following criteria will be excluded: patients with (1) inability to exercise or other diseases that may affect their physical activity; (2) cognitive disorder; (3) serious comorbidities or complications, such as heart failure, autonomic neuropathy, and recent stroke; (4) a high risk of falling; (5) a history of cancer; or (6) end-stage renal disease dialysis.

### Random assignment and blinding

After the baseline assessment, we will randomly assign the participants into three equal groups (1:1:1), namely, the HIIT group, MICT group, and guideline group. This study is an assessor-blind randomized controlled trial, and indicators will be measured at baseline and after 12 weeks by assessors blinded to group allocation. Study interventions will be delivered by study staff. A randomization table will be provided by the Department of Statistics, School of Public Health, Fudan University (Excel software; Microsoft). Study staff who blinded to the group allocation will collect the measurements and maintain possession of the randomization table. The group allocation of participants will be recorded on a paper in an opaque envelope that will only be opened after the baseline assessment of participants has been completed.

### Sample size calculation

We assumed MICT is superior to guideline-based group in glucose control and cognitive function. Moreover, we also test whether HIIT was superior to MICT group. Limited studies have compared the HIIT or MICT and guideline groups on HbA1c or BDNF. We assumed a glycosylated hemoglobin (HbA1c) mean (SD) difference in change of 0.2 (0.3)% between MICT groups and guideline groups. Based on previous study (Terada et al., 2013), an additional mean (SD) difference in change of HbA1c of 0.2 (0.3)% between HIIT and MICT was assumed. The mean difference (SD) in BDNF levels was assumed to be 1.2 (1.4) ng/ml between MICT and guideline group. Additional 1.2 (1.4) ng/ml for BDNF between HIIT and MICT was assigned (Kovacevic et al., 2020). We will adopt the larger sample size to ensure adequate power. Finally, we intend to enroll 330 participants (110 per group) with a power of 90%, a one-sided α of 0.025 and an expected dropout rate of 20%. The α value of 0.025 will be equal assigned to HbA1c and BDNF. PASS software (version 15.0, NCSS Statistical Software) was used to calculate sample size. If the upper confidence interval of the MD in HbA1c levels between the HIIT or MICT interventions and the reference group falls below zero (<0.0%), HIIT or MICT is considered superior to the reference group in terms of improving glucose control. When the lower confidence interval > 0.0% of the MD in BDNF, it would be declared superior to the reference group for improving cognitive function.

### Intervention

All patients will be advised (50 min seminar) on the health benefit of exercise according to current guidelines before intervention. We report our intervention according to the TIDieR checklist. After the informed consent and screening steps are completed, patients will be randomly assigned to three groups (in a 1:1:1 ratio): the MICT group, involving moderate-intensity continuous training (MICT); the HIIT group, involving high-intensity interval training (HIIT); and the guideline group, involving guideline-based physical activity (Figure 1). Participant compliance is of paramount importance for this study, and every possible measure will be taken to maintain adherence to the exercise intervention and follow-up plans. If a participant fails to attend three consecutive sessions, the researcher will actively inquire about the reasons for their absence and provide encouragement for continued participation. To account for the minimal effective dose of exercise, a minimum attendance rate has been established based on previous research findings (Abreu et al., 2021). Specifically, participants who complete fewer than 24 training sessions will be classified as dropouts. The adherence to the training program will be meticulously monitored and regularly documented throughout the trial, allowing for comprehensive analysis and evaluation. Reasons for terminating the test: if serious adverse events occur during exercise, the test should be terminated in time. Adverse events: (1) severe hypoglycemia and complications: shaking, dizziness, abnormal sweating, headache, blurred vision, (2) chest pain, arrhythmia, (3) breathing difficulties and asthma.

#### Guideline group

Participants in the guideline group will perform 1-time supervised 40–50 min of dedicated training (including jogging and brisk walking) 3 times a week in addition to daily physical activity, and the rate of perceived exertion (RPE) will be set at 3–4 (Borg RPE scale: 12–16). Afterward, WeChat–a mobile-based messaging App (Tencent cop., China)- will be used to remotely monitor and supervise exercise weekly. The exercise data (intensity and duration) will be collected weekly to ensure sufficient exercise time and to encourage exercise motivation.

### Training intervention

Participants in the MICT and HIIT groups will perform exercise of different intensity on a treadmill. Exercise intensity, duration, the heart rate (HR), and blood pressure will be recorded before and after exercise, along with any adverse events during exercise. To minimize the risk of bias in the interpretation of the results, we matched the amount of exercise between the HIIT and MICT groups based on workload. Exercise training will be terminated at signs of any serious adverse event (SAE), such as, severe hypoglycemia and complications: shaking, dizziness, abnormal sweating, headache, blurred vision; chest pain, arrhythmia; breathing difficulties and asthma; pain and fatigue.

#### HIIT group

Participants will perform training with supervision, involving walking on a flat or inclined treadmill 3 times a week. The HIIT protocol consists of a 10-min warm-up (at 50–60% of the peak HR), 10 × 1-min intervals at 85–95% of the peak HR separated by 1-min periods of active recovery (at 60–70% of the peak HR), and a final 5-min cooldown, which corresponds to the Borg RPE scale of 16–17. An HR monitor (Polar Electro, Kempele, Finland) will be used to obtain the training HR. The speed and incline of the treadmill will be continuously adjusted to ensure that each training session is performed at the prescribed HR.

#### MICT group

Similar to the HIIT group, the participants in the MICT group will perform training with supervision, involving walking on a flat or inclined treadmill 3 times a week. Corresponding to the amount of exercise (exercise intensity × duration) of the HIIT sessions, each MICT session will consist of a 5-min warm-up period and a 5-min cooldown period at 30% of the peak HR, separated by 35 min of exercise at 65–75% of the peak HR. An HR monitor (Polar Electro, Kempele, Finland) will be used to obtain the training HR. The speed and incline of the treadmill will be continuously adjusted to ensure that each training session is performed at the prescribed HR.

### Measurements

The research assistants who blinded to group allocation will collect baseline data from the patients, including age, course of the disease, height, weight, blood pressure, smoking history, socioeconomic status, time since retirement, and history of antidiabetic medication. These assessments will provide a comprehensive overview of the participants’ characteristics. The primary outcome measures of the study will be the HbA1c and BDNF levels. Additionally, the secondary outcome measures will include physical activity levels, anthropometric parameters (weight, waist circumference, hip circumference, and BMI), physical parameters (including fat percentage, muscle percentage, and the fitness rate), cardiorespiratory fitness indicators (blood pressure, the heart rate, vital capacity, and maximum oxygen consumption), biochemical indicators (high-density lipoprotein, low-density lipoprotein, triglyceride, total cholesterol, and HbA1c), inflammation level (C-reactive protein), cognitive function (assessed through reaction time and dual-task gait test performance), and motor function (evaluated through static balance, dynamic balance, single-task gait test performance, and grip strength). To collect these data, participants will be assessed before the start of the intervention and again after the 12-week intervention. Notably, all exercise tests and data collection will be conducted by research assistants blinded to patient group allocation.

### Primary outcomes

#### Glycosylated hemoglobin

To measure HbA1c levels, venous blood samples will be collected in the morning from participants in a fasting state, both before and after the 12-week intervention period. The blood samples will be gently mixed by inverting them multiple times and then allowed to stand at room temperature for 2 h. Afterward, the samples will be centrifuged at 3,000 rpm for 5 min at 4°C. The determination of HbA1c content will be conducted using a fully automated HbA1c analyzer through high-performance liquid chromatography. Each index will be recorded twice to ensure accuracy, and the average value of the two measurements will be used for statistical analysis. In this study, a HbA1c change of 0.2% will be considered the minimum effect size that would have clinical significance and would be of interest in comparisons between the HIIT or MICT groups and the guideline group. HbA1c is an excellent indicator for long-term blood glucose level for 3 months.

### Brain-derived neurotrophic factor

Fasting participants will provide blood samples before and after the 12-week exercise intervention to measure serum BDNF levels. The analysis of serum BDNF levels will be performed using an enzyme-linked immunosorbent assay (ELISA) kit in accordance with the instructions provided by the manufacturer. BDNF is considered an indicator of neuroplasticity and cognitive function, and decreased BDNF levels have been associated with cognitive (Zhen et al., 2013).

### Secondary outcomes

#### Physical activity test

We will employ the Actigraph GT3X (USA), a triaxial accelerometer operating at a frequency of 30 Hz with a 10-s interval, to monitor participants’ physical activity. Prior to the test, we will thoroughly explain the purpose, requirements, and procedures to the participants. The accelerometers will be worn on the right anterior superior iliac spine for a week, except during activities involving water, such as bathing and swimming. Throughout the monitoring period, participants will wear the accelerometers continuously, except during water-related activities, for a minimum of 10 h per day, spanning five working days (Monday to Friday) and two rest day (Saturday and Sunday), ensuring the collection of valid data. To assess physical activity, various parameters will be measured, including average metabolic equivalents (METs) and sedentary time. Data analysis will be conducted using ActiLife 5.0 analysis software.

### Blood pressure (BP) and heart rate

BP will be measured using the Accoson sphygmomanometer (Accoson, Ayrshire, UK), and HR will be measured using the Omron HEM 3 automated blood pressure machine (Omron, Osaka, Japan). Doctors will conduct the tests while participants maintain a seated position.

### Cardiorespiratory fitness

Vital capacity will be tested using an electronic spirometer. Cardiopulmonary exercise testing (CPET) will be performed with a cycle ergometer (Ergoline, Germany), and a ramped test protocol of 15 W/min at 60 ± 5 rpm, continued until volitional exhaustion. A 12-lead electrocardiogram (ECG) device will provide continuous monitoring during the test, and respiratory gas analysis will be assessed in a breath-by-breath manner with a computerized metabolic cart (Schiller CS-200 Ergospirometry, Switzerland). The ventilatory anaerobic threshold (VO_2_AT) will be assessed using the V-slope method by two independent clinicians. In cases where the VO_2_AT deviation is over 10% between the two clinicians, a third clinician will be asked to provide an additional assessment of the VO_2_AT. An average of the two nearest values will be selected. VO_2_peak will be recorded as the average value of VO_2_ during the final 30 s of the test and expressed in ml/kg/min. Cardiopulmonary exercise testing (CPX) will be performed at baseline and at the end of 12 weeks. CPET will be repeated using the same protocol as the baseline test and performed at the same time of day. Cardiorespiratory fitness is an objective measure of physical fitness and has been recognized as protective indictor against cardiovascular events, T2DM and all-cause death.

### Anthropometric parameters

A Seca 213 stadiometer (Seca; Hamburg, Germany) will be used to measure height and weight, and tape will be used to measure the waist and hip circumference. Finally, the BMI (kg/m^2^) of participants will be calculated using the following formula: body weight (kg)/[(body height*body height)m^2^]. BMI is a widely used indictor for obesity and risk factor for cardiovascular events in patients with T2DM.

### Percent body fat

An Inbody 770 (Inbody Co., LTD., Seoul, Republic of Korea) scan will be used to conduct body fat tests. The Inbody 770 device has reliability and validity for body composition assessment (Poblete Aro et al., 2015). Participants will stand barefoot on a platform with electrodes and undergo measurement through hand-held handles. Finally, the body fat percentage of participants will be calculated using the following formula: body fat weight/total weight. There is a significant association between body fat percentage and the risk of cardiovascular events in patients with T2DM.

### Blood glucose, blood lipids and C-reactive protein

During the study, blood samples will be collected from participants before and the 12-week exercise intervention. Blood collection will be performed in the next morning after overnight fast. Four milliliters of venous blood will be drawn, thoroughly mixed by shaking, and allowed to stand at room temperature for 2 h. Subsequently, the blood will be centrifuged at 4°C and 3,000 rpm for 5 min. The resulting supernatant will be stored in a refrigerator at −70°C. To assess various parameters, including glucose, high-density lipoprotein cholesterol (HDL-C), low-density lipoprotein cholesterol (LDL-C), triglyceride (TG), total cholesterol (TC), and C-reactive protein (CRP), a fully automated analyzer (7,071°) will be utilized. Each index will be measured twice, and the average value will be used for statistical analysis. Fasting blood glucose is an indicator for blood control during the night. Similarly, blood lipid levels are strong risk factors for incidence of cardiovascular events, regardless presence of T2DM.

### Reaction time

Before the test, the entire testing process will be explained by researchers. Response times reflect cognitive function, impaired response times is an indicate of impaired daily function in patients with T2DM (Wong et al., 2014). During the test, the participants will press the start button and wait for the signal to be sent. When any signal is presented, the participants should press the button as fast as possible. After the signal disappears, the participants should press the start button again and wait for the next signal to be presented, for a total of 5 signals. After the participants complete the fifth signal response, the signal key will emit a prompt sound, which indicates the end of the test. There will be three tests in total, and the best score will be recorded.

### Single- and dual-task gait tests

Before conducting the test, the researcher will explain the specific requirements of the single- and dual-task gait tests and allow the participants to practice on the Footscan gait system (RsScan International, Olen, Belgium). Our previous study demonstrated the reliability and validity of the Footscan gait system in testing gait function in the elderly population (Yan et al., 2022). Gait can reflect motor and cognitive function in patients with T2DM, and the decline in dual-task gait test performance was significantly associated with motor and cognitive dysfunction in patients with T2DM (Herings et al., 2022). During the formal test, participants will be instructed to walk at their normal pace for a total of five trials. Following a brief break, a dual-task gait test will be conducted, in which participants will be instructed to walk at their normal pace while simultaneously performing addition and subtraction tasks aloud, with numbers ranging up to 100. The results obtained will be based on the average of the five trials conducted. The main parameters measured during the single- and dual-task gait tests will include gait velocity, stride length, stride width, and gait cycle. These parameters provide valuable insights into the participants’ walking abilities and help to identify any potential changes or differences between single- and dual-task walking conditions.

### Grip strength

Grip strength will be measured using a grip strength dynamometer. As a convenient test, grip strength has been shown to reflect muscle quality (Boonpor et al., 2021). Participants will be instructed to assume a standing position with their arms fully extended. They will be asked to maintain a firm grip on a dynamometer, with the size of the grip adjusted to accommodate their palm size. Three measurements will be taken for each hand to ensure accuracy and reliability. To prevent any potential errors, the dynamometer will be cleared before each test. This will ensure that any residual tension or previous measurements do not influence the results. The maximum grip strength achieved during the three measurements for each hand will be recorded as the final maximum grip strength value for that hand.

### Static balance

Static balance function will be assessed by instructing the participant to stand on one foot with their eyes closed. Impaired static balance is a potential risk factor for fall (Dixon et al., 2017). During the test, participants will be instructed to lift one foot, and the tester will start the timer. The test will continue until the supporting foot moves or the raised foot lands on the ground, indicating the end of the trial. Both legs will be tested three times each to determine the maximum value achieved. To ensure accurate results, a 30-s break will be provided between each test. This break serves to minimize the effects of fatigue and other factors that could influence performance.

### Dynamic balance

The Y balance test (YBT) will be used to test the dynamic balance function of the participant. Our previous study demonstrated the reliability and validity of the YBT in testing dynamic balance function in the elderly population (Yan et al., 2022). Decreased dynamic balance function static balance is a potential risk factor for fall (Dixon et al., 2017). Prior to the commencement of the YBT, participants will receive clear instructions and engage in practice sessions to ensure their understanding and proficiency in performing the test. During the test, participants will stand barefoot and begin by placing their dominant leg on a support board, with their hands at their waist. They will aim to maintain their balance while exerting force with the non-supporting leg to push the indicator as far as possible. Subsequently, they will carefully return their body back to the initial test position. The same procedure will be repeated with the non-dominant leg. To obtain accurate and reliable results, each participant will perform three trials with each leg. The average of the three trials for each leg will be used for subsequent analysis. To account for the influence of participants’ height on the test results, the YBT data will be normalized. Normalization will be achieved by calculating the ratio of the extension distance to the leg length distance and multiplying it by 100%. By normalizing the YBT data, we can effectively account for the individual’s height and obtain a standardized measure of their performance. This allows for meaningful comparisons and analysis across different participants.

### Statistical analysis

The intention-to-treat principle will be employed for all analyses, ensuring that participants are analyzed according to their assigned treatment groups. Linear mixed models will be utilized, incorporating participant and baseline values as random effects, to estimate changes within groups (with a difference between groups of *P* < 0.1) for all outcome measures. To address missing data, the multiple-imputation method will be employed to test its influence on the analysis. All statistical analyses will be conducted using R software (version 4.0.2, the R Project for Statistical Computing^2^). The hypothesis tests will be two-sided, and statistical significance will be defined as *p* < 0.05. For the primary outcomes, multiple will be not necessary as the α value have been allocation in the sample calculation. Importantly, no adjustments for multiple comparisons will be made for secondary outcomes, and therefore, their interpretation should be considered exploratory in nature. Subgroup analyses based on sex, duration of T2DM, and BMI will also be performed to examine potential variations within these subgroups. A per-protocol sensitivity analysis will be applied.

## Discussion

The increasing prevalence of sedentary behavior among elderly individuals with T2DM has become a substantial public health concern, requiring effective prevention and management strategies. A sedentary lifestyle in this population is associated with cardiovascular risks, cognitive decline, and motor dysfunction. Regular exercise is highly beneficial for sedentary elderly patients with T2DM due to its ability to counteract the hazards of a sedentary lifestyle (Safdar et al., 2010). Despite the acknowledged benefits of regular exercise, motivating sedentary elderly patients with T2DM to engage in consistent exercise remains a challenge. Therefore, it is crucial to identify an optimal exercise regimen for this population, considering factors such as exercise motivation and duration. However, the interaction between exercise intensity and duration makes it difficult to establish an optimal dose-response model for exercise. A key aspect of cardiac rehabilitation is comparing HIIT with MICT, as HIIT is considered a time-efficient alternative to MICT and thus facilitates adherence (Heinrich et al., 2014). While several published studies have demonstrated the cardioprotective effects of HIIT in patients with T2DM through improved glycemic control and long-term cardiometabolic adaptations, this evidence remains insufficient for sedentary elderly patients with T2DM (De Nardi et al., 2018; Mendes et al., 2019; Suryanegara et al., 2019). Based on existing research, it is reasonable to hypothesize that HIIT, characterized by higher exercise intensity, may lead to the excess post-exercise oxygen consumption and stimulate greater endogenous responses to yield benefits (Li et al., 2022).

This greater endogenous stimulation also induces greater cardiorespiratory fitness; since maximal oxygen consumption (VO_2_ max) reflects the ability of the cardiorespiratory system to support physical performance at the highest workload by ensuring oxygen delivery to skeletal muscle, there may be a positive correlation between cardiorespiratory function and skeletal muscle function (Koh et al., 2018). Improved cardiorespiratory function and skeletal muscle function are closely associated with increased overall metabolic levels, and elevated overall metabolic levels may enhance glucose metabolism.

Current evidence regarding HIIT-induced improvements in motor function primarily comes from skeletal muscle adaptations, while conclusions regarding overall motor function in older patients with T2DM are lacking. Sedentary elderly patients with T2DM exhibit worse muscle function and have a higher risk of falls (due to slower gait speed and poor balance control). Therefore, we evaluated the effects of HIIT and MICT on muscle strength and overall movement (dynamic and static balance and gait) in sedentary elderly patients with T2DM.

To date, the specific effects of HIIT and MICT on cognitive function in elderly sedentary patients with T2DM remain unknown. Most studies investigating the impact of HIIT on cognitive function have been conducted in healthy older individuals or animal models. These studies have revealed divergent effects of HIIT and MICT on different aspects of cognitive function. Regarding overall cognitive function, MICT has been reported to provide the greatest benefit in terms of executive function, whereas HIIT has yields greater improvements in information processing speed (Coetsee and Terblanche, 2017). Importantly, cognitive impairment in patients with T2DM manifest in a variety of ways, including reduced levels of cognitive markers such as BDNF, decreased information processing speed, and impaired performance during the dual-task gait test (Silsupadol et al., 2009; Kovacevic et al., 2020; Novak et al., 2022). Given these considerations, we evaluated the effects of HIIT and MICT on elderly sedentary patients with T2DM from multiple perspectives, including cognitive markers, information processing speed (reaction times), and movement-cognition interaction (the dual-task gait test).

This trial is subject to several limitations that should be acknowledged. First, this study will not include an in-depth analysis of metabolic marker alterations in response to exercise in patients with T2DM. Future research could incorporate metabolomics to investigate changes in cardiovascular markers in response to exercise. Second, we will not conduct a comprehensive investigation into motor and cognitive functions. Future studies could employ neuroimaging tools to assess changes in cognitive-related cortical activity and functional connectivity. Additionally, improving the detection accuracy is crucial. Assessment of dynamic cortical activity data during exercise could provide valuable insights into the cortical mechanisms underlying changes in motor function, and muscle strength testing could be enhanced through the use of objective equipment such as isokinetic devices. Furthermore, the study population will consist of only Asian individuals. Racial and regional differences may limit the generalizability of the findings.

## Conclusion

In conclusion, the findings of this trial will have the potential to contribute to the identification of an appropriate exercise regimen that can effectively enhance cardiometabolic health, motor function, and cognitive function in elderly sedentary patients with T2DM. The results will have important clinical implications for the development of personalized exercise prescriptions tailored to the specific needs and goals of individuals within this population. By evaluating the effectiveness of HIIT and MICT for various aspects of health and functioning, including cardiovascular health, motor function, and cognitive function, healthcare professionals can optimize exercise interventions and improve the overall wellbeing of elderly sedentary patients with T2DM.

## Data availability statement

The raw data supporting the conclusions of this article will be made available by the authors, without undue reservation.

## Ethics statement

The studies involving human participants were reviewed and approved by the Ethics Review Committee of the Reproductive Hospital Affiliated with China Medical University (approval number: 202203). The patients/participants provided their written informed consent to participate in this study.

## Author contributions

ZY, WL, and XL conceptualized the study, supervised the research, acted as a guarantor, and prepared the first draft. PY helped design the study. XL and ZY revised the manuscript. XL performed statistical calculations. ZY and PY provided research funding. All authors contributed to the manuscript and approved the final version.

## Footnotes

1
https://www.chictr.org.cn/showproj.html?proj=172164

2
http://www.r-project.org
